# Quantitative evaluation of the site-dependent cell viability in three-dimensional hepatocyte spheroids based on dynamic optical coherence tomography

**DOI:** 10.1117/1.JBO.30.3.035003

**Published:** 2025-03-14

**Authors:** Ling Wang, Chang Wang, Qiansen Li, Rongzhen Fu, Chen Xu, Mingen Xu

**Affiliations:** a Hangzhou Dianzi University, Automation College, Hangzhou, China; b Zhejiang Provincial Key Laboratory of Medical Information and Biological 3D Printing, Hangzhou, China

**Keywords:** spectral-domain optical coherence tomography, dynamic optical coherence tomography, hepatocyte spheroids

## Abstract

**Significance:**

Hepatocyte spheroids (HCSs) are three-dimensional (3D) *in vitro* models that exhibit a multilayered structure with site-dependent cell viability. The non-invasive identification of HCS structure and viability variation is essential in fully exploiting the potential of HCS as a model for liver disease research.

**Aim:**

We aim to achieve long-term, non-invasive monitoring and quantification of HCS cell viability based on dynamic optical coherence tomography (D-OCT) and enhance visualization of HCS internal activity with D-OCT pseudo-color images.

**Approach:**

We employed D-OCT based on power spectrum analysis with an appropriate optical coherence tomography time-series image acquisition rate to obtain the motion frequency distribution of cells within HCS, thus distinguishing and segmenting the viable and necrotic cell layers based on the average frequency of cellular activity, and quantify the tissue activity using the pixel ratio of the segmented viable region to the total spheroid region. Meanwhile, we used the hue saturation value color mapping method to enable enhanced visualization and high-precision segmentation of viable and necrotic cell layers in HCS.

**Results:**

The feasibility of the D-OCT method was verified experimentally with three sets of HCS samples (HCS-2000, HCS-5000, and HCS-10000) by comparison with a confocal laser scanning microscope. The cells in C3A-HCS were found to be active mainly in the range of 8 to 13 Hz by D-OCT detection. 3D D-OCT pseudo-color images of HCS with a maximum diameter of 450  μm were displayed, and the 3D structures of necrotic and viable cell layers were identified by mask segmentation based on the average cell activity frequency threshold (10.5 Hz). The longitudinal necrotic process of three sets of HCS samples with differing inoculated cell numbers was monitored and quantified over 29 days.

**Conclusions:**

The employed D-OCT method can be used to quantitatively evaluate the site-dependent cell viability in HCS and possesses the potential for long-term, non-invasive monitoring and quantification of HCS viability.

## 1 Introduction

The liver is the primary organ involved in the metabolism of toxins in the human body, and hepatocytes are the primary functional cells responsible for maintaining liver function. Hepatocytes can be used to construct two-dimensional (2D) and three-dimensional (3D) *in vitro* hepatocyte models to assess hepatotoxicity.^1^^,^^2^ Compared with monolayer 2D cell culture models, the 3D hepatocyte spheroid (HCS) model simulates the liver microenvironment with cell physiology and function, which is beneficial for toxicity testing and drug screening.^3^ The multilayered structure of highly viable cell layers, low active cell layers, and necrotic cell layers would occur in the HCS.^4^^–^^6^ As the presence of necrotic cells in the central region of HCS distorts the toxicity results,^4^ various methods have been employed to detect the overall viability of 3D cell spheroids, including the necrotic cell layers in the core region. Currently, fluorescence microscopy,^7^ confocal laser scanning microscopy (CLSM),^8^^,^^9^ adenosine triphosphate (ATP),^10^^,^^11^ and 3-[4, 5-dimethylthiazol-2-yl]-2, 5-diphenyltetr-azolium bromide (MTT)^12^ methods are widely used to assess cell viability in 3D cell spheroid models. However, the use of exogenous markers or disruption of the spheroid structure prevents the continuous cultivation of HCS and the longitudinal study of tissue activity changes. In addition, the large diameter and dense multilayered structure of HCS result in uneven penetration of staining reagents, causing intrinsic fluorescence intensity errors in imaging, and affecting the accurate measurement of cell viability at the center of the HCS.^6^^,^^13^ Therefore, a non-destructive, non-invasive method with a large penetration depth is crucial to detect the cross-sectional tissue activity and the overall cell viability of HCS.

Optical coherence tomography (OCT) as a non-destructive, non-contact 3D imaging technology based on the principle of low-coherence interference^14^ features a large field of view (10×10  mm) and deep imaging penetration (1 to 3 mm), enabling the detection of the internal microstructure of the entire HCS.^7^ Currently, OCT has been applied to 3D structural imaging, longitudinal morphological monitoring, volume quantification, and other aspects of cellular spheroids.^7^^,^^9^^,^^14^^–^^18^ However, the traditional OCT imaging method fails to characterize tissue dynamics information related to cell viability. Upon apoptosis or necrosis of healthy cells, the dynamic information of cells (motion amplitude and frequency) undergoes substantial changes.^19^ Using cell motility as an endogenous contrast agent allows the non-destructive detection of tissue dynamics information of the sample.^20^ Park et al. used dynamic full-field OCT (D-FFOCT) to detect the dynamic activity of HeLa cells under different viability conditions, demonstrating that healthy cells exhibit a lower mean frequency and a larger activity range compared with dead cells.^19^ Dynamic optical coherence tomography (D-OCT) was initially employed in time-domain full-field OCT. However, an array of shortcomings, such as a small imaging field of view, shallow penetration depth, and the inability of axial cross-layer imaging, make it difficult to meet the overall imaging requirements of thick tissue samples such as HCS.^21^^,^^22^ Practical applications show that D-OCT would overcome the shortcomings of D-FFOCT and detect cellular dynamics information of the entire cross-section of thick tissue samples.^23^^–^^25^ Existing dynamic processing methods include the standard deviation (STD),^19^^,^^26^^,^^27^ log intensity variance (LIV),^24^^,^^28^ OCT correlation decay speed (OCDS),^25^ and power spectral density (PSD).^19^^,^^26^^,^^27^^,^^29^^–^^32^ Among them, STD and LIV are used to quantify the amplitude of tissue dynamics, whereas the OCDS can discern the speed of tissue dynamics. These methods can all enhance the dynamic contrast in OCT with around 10 repeated frames. Some researchers have integrated the PSD over three different frequency bands, such as 0 to 0.5 Hz, 0.5 to 5 Hz, and 5 to 25 Hz, to obtain three coefficients describing the frequency-dependent variation in signal intensity.^26^^,^^30^^–^^32^ Although the PSD algorithm requires over 100 repeated frames for accurate PSD measurements, it provides more comprehensive tissue dynamics information. Moreover, the PSD method intrinsically has better noise resistance capability due to its frequency-domain analysis nature. Thus, the mean frequency method, as one of PSD methods, can be a promising tool in the quantitative evaluation of the site-dependent cell viability in HCS.

In this study, we employed a D-OCT imaging method based on spectral domain-OCT (SD-OCT) and power spectrum analysis of the time series of OCT intensity images, which can visualize the site-dependent cell activity frequency for the quantitative analysis of cell viability inside HCS quickly and accurately. We performed D-OCT imaging on fully viable C3A-HCS and fully necrotic C3A-HCS, observing significant differences in the mean frequency between the two and validated the D-OCT imaging results using CLSM to confirm their accuracy in distinguishing cell viability. We also demonstrated the site-dependent active motion of cellular structure in 2D and 3D for the HCS samples and segmented the 3D structures of the highly active cell layers and the necrotic cell layers. Furthermore, the longitudinal necrotic process of three sets of HCSs with different cell inoculation numbers was visualized and quantified for 29 days. We demonstrated that the utility of the D-OCT imaging method would facilitate the quantitative evaluation of the site-dependent cell viability in 3D hepatocyte spheroids for advanced liver disease research.

## 2 Methods

### 2.1 Preparation of Hepatocyte Spheroids

HCSs used in the experiment were obtained from Hangzhou Regenovo Biotechnology Co., Ltd., and prepared using a liquid overlay technique on concave-bottomed culture wells with low adhesion.^33^^,^^34^ Briefly, C3A cell line authentication and mycoplasma testing were also performed by Regenovo. $10^{5}\text{ cells}/\mathrm{ml}$ concentrations of C3A hepatocyte suspensions (C3A-HCS) and 20  μl (HCS-2000), 50  μl (HCS-5000), and 100  μl (HCS-10000) volumes were inoculated. The surface tension of the concave surface prevented cell adhesion and facilitated cell aggregation into tightly arranged microspheres to form C3A-HCS. The 96-well plate containing C3A-HCS was placed in a dedicated incubator at a constant temperature of 37°C and 5% ${\mathrm{CO}}_{2}$ concentration, replacing the specialized HCS culture medium (Live™ Medium/M, Regenovo Co. Ltd., Hangzhou, China) every 48 h. Before each OCT imaging, the plate was sealed with a sterile sealing film to ensure the retention of an adequate amount of ${\mathrm{CO}}_{2}$ and the maintenance of C3A-HCS activity during OCT detection.

### 2.2 Experimental Protocols and Devices

**Fig. 1:**
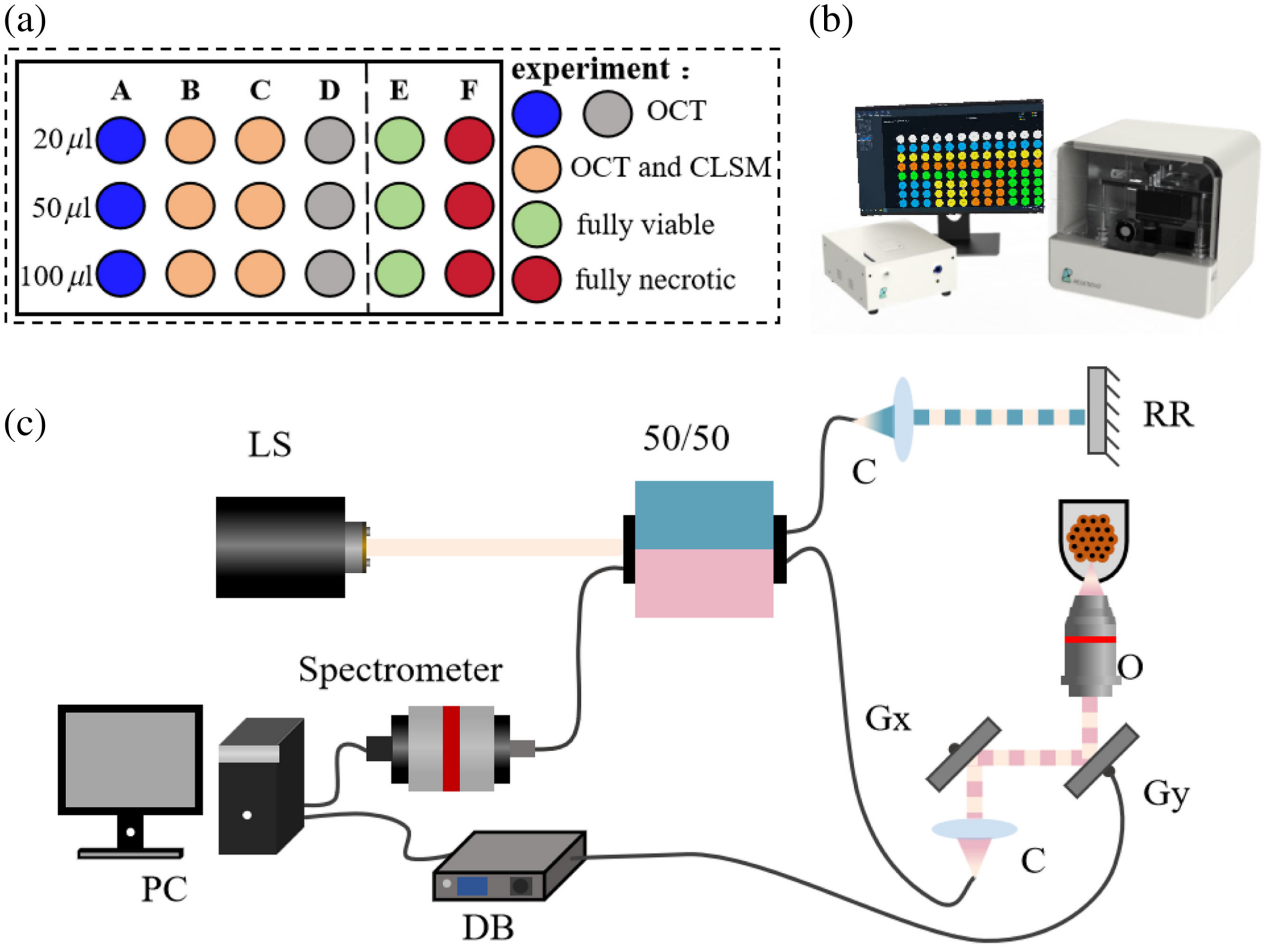
Illustration of experimental devices and group scheme. (a) Schematic diagram of the experimental groups. (b) Product rendering image of SD-OCT system. (c) Schematics of the SD-OCT setup. LS, light source; 50/50, 50/50 fiber coupler; C, collimators; RR, retroreflector; Gx and Gy, galvanometer mirror scanners; PC, computer for data acquisition and scanning control; DB, driver board; O, objective.

In this paper, the feasibility and effectiveness of dynamic OCT to detect the site-dependent activity of C3A-HCS were studied by interactive verification experiment with CLSM, 3D dynamic OCT imaging, and dynamic OCT continuous monitoring experiment. The experiment was carried out in three batches, each using two 96-well plates. Each plate contained three types of cell spheroids (HCS-2000, HCS-5000, and HCS-10000). The first plate consisted of 12 spheroids divided into four groups (A, B, C, and D), whereas the second plate contained six spheroids divided into two groups (E and F). In each batch of the imaging experiment, the six groups of C3A-HCS were subjected to different experiments [Fig. 1(a)]. For group A (blue wells), the C3A-HCS were monitored continuously with D-OCT for up to 1 month to observe the longitudinal necrotic process of C3A-HCS with different cell seeding numbers. For groups B and C (orange wells), D-OCT and CLSM imaging were conducted on days 10 and 29, respectively, to verify the correspondence between D-OCT and CLSM results. Group E (green wells) consists of C3A-HCS on the first day of spheroid formation, which we consider fully viable, whereas group F (red wells) consists of C3A-HCS fixed with 4% PFA for 10 min, which we consider fully necrotic. D-OCT and CLSM imaging were performed on groups E and F to validate D-OCT’s ability to distinguish between live and dead cells. To minimize the impact of the external environment on the cell viability of HCS, fluorescence staining and microscopic imaging were performed immediately after the central section of OCT detection. The C3A-HCS samples in group D (gray wells) that served as backups were used for 3D dynamic OCT imaging research at the right time. The aforementioned six groups of experiments were repeated for three batches, and to improve the experimental efficiency, the three-batch experiments were performed with a total of 6 well plates and 54 cell spheres, and each well plate was sampled at an initial testing interval of 3 days.

The SD-OCT system (Bio-architect, Regenovo, Hangzhou, China) was employed for OCT intensity image acquisition as described in our previous work.^35^ In short, it used a broadband light source with a central wavelength of 1310 nm and a full-width half-maximum of 248 nm, along with a microscope objective of 0.2 NA (LSM03, Thorlabs, Newton, New Jersey, United States), a line-scan camera with a resolution of 2048 pixels, and a line scan rate of up to 76 kHz (GL2048L, Sensors Unlimited, Princeton, New Jersey, United States); the axial resolution (in air) was measured to be 3.5  μm, and the lateral resolution was 13  μm. To ensure the accuracy of image dimensions, the refractive index of HCS was calculated according to the refractive index of biological tissues (1.38). Thus, the axial resolution in the sample is 2.53  μm, with a maximum imaging depth of 2.6 mm, enabling full-depth 3D imaging of the HCS. Figure 1(c) shows the schematics of the SD-OCT setup. Figure 1(b) shows the product rendering image of the SD-OCT system. During the imaging process, the inverted OCT probe was positioned directly below the supported sample stage, and the 96-well plate containing the C3A-HCS and culture medium was placed on the sample stage. The 96-well plate was moderately tilted to minimize the specular reflection of the concave-bottomed culture wells, and the focusing depth was adjusted to the middle position of the C3A-HCS to ensure a uniform OCT intensity signal.

### 2.3 Dynamic OCT Imaging Method

In this report, the raw data of dynamic OCT imaging were obtained from the time-series images captured by the SD-OCT system, and the power spectrum changes in OCT intensity caused by the activity of viable cells were analyzed and normalized to calculate the mean frequency of cell motions. Real-time video (see Video 1) of the OCT intensity image of C3A-HCS revealed that the activity frequency of viable cells in the outer region of C3A-HCS was relatively low, but the activity magnitude was higher. Appropriate parameter settings were required for D-OCT signal acquisition. First, the scanning range, pixel size, and the location of each B-scan were set. To better compare with CLSM, the B-scan image requires the maximum cross-section of HCS. The differences in the number of seeded cells and culture days resulted in different HCS diameters ranging from 100 to 500  μm. To obtain a complete cross-sectional image of the HCSs, we both set the axial and lateral pixel size of the B-Scan to 2.53  μm, and each B-scan is composed of 660 A-scans, with each A-scan comprising 1024 pixels, generating a field of view of the B-Scan image at 1.67  mm (x)×2.59  mm (z). To reduce image processing time, we crop the B-scan image to a resolution of 660 (x)×660 (z) pixel, corresponding to a field of view of 1.67  mm (x)×1.67  mm (z). Second, the A-scan scanning rate was set. According to Shannon’s theorem, the frequency range of cellular activity that D-OCT can detect is half of the B-scan scanning frequency.^36^ As the movement frequency of C3A cells in HCS is below 20 Hz, the sampling rate for dynamic OCT needs to be set higher than 40 Hz. With an A-scan scanning frequency of 48 kHz, the ideal B-scan imaging time is 13.75 ms. However, due to the delay in data storage and transmission, the actual B-scan acquisition speed was 56  frames/s (fps), that is, the actual time resolution is 18 ms, and the corresponding detectable motion frequency range is 0 to 28 Hz. Next, we set the scanning repetition times for the same x−z cross-section position, from where a time series of 512 OCT frames was collected in 9.2 s. Finally, 3D data acquisition was carried out based on 2D data acquisition. 2D images of the x−z cross-sections (B-scan) were collected at equal intervals along the y-axis for the entire spheroid. Considering the isotropic properties of the 3D resolution of the image data, the sampling interval among adjacent B-scans was set to 2.53  μm to ensure the quality of subsequent dynamic 3D image reconstruction.

**Fig. 2:**
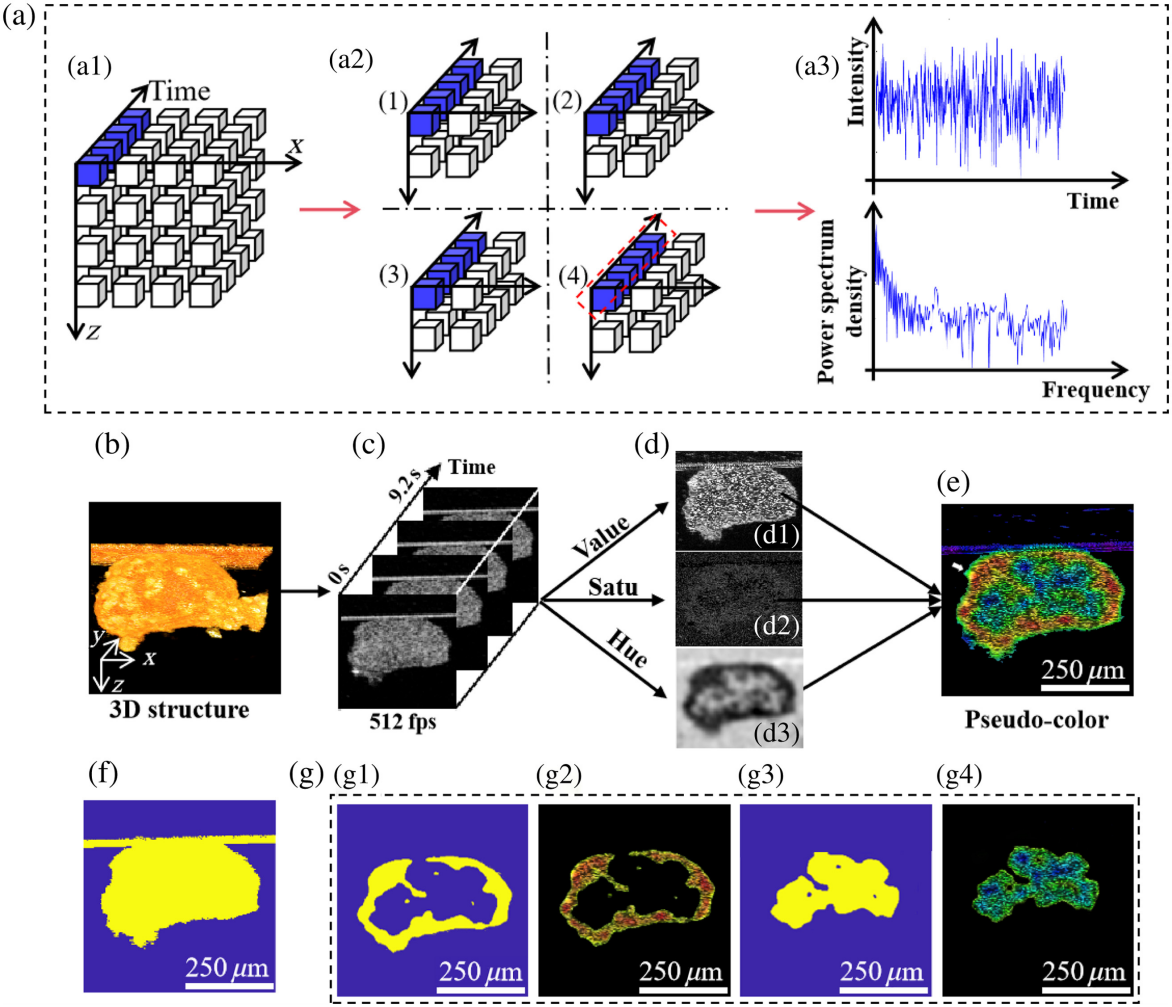
Accelerated calculation method and pseudo-coloring of the D-OCT image and creation of the segmentation mask for viable and necrotic cell layers. (a) Parallel computation process: (a1) 3D cube constructed from the time-series intensity data of a single x−z cross-section, (a2) quartered 3D cube used for the parallel calculation of mean frequency, and (a3) time-dependent signal intensity of a single pixel in the OCT image and the corresponding power spectrum information. (b) 3D reconstruction of the C3A-HCS structure. (c) Time-series OCT intensity image at the middle cross-section of C3A-HCS. (d) Calculated STD (value), $f_{\mathrm{STD}}$ (Satu), and $f_{\text{mean}}$ (hue) images. (e) Pseudo-color image. (f) Segmentation mask for the HCS cross-section. (g) Segmentation masks for different activity areas and corresponding segmentation results of the pseudo-color image. The white arrows point to the misclassified areas removed using the masking method, which we discuss in Sec. 4.

The collected time-series B-scan images were processed as follows: first, each frame was normalized by its respective mean intensity to eliminate the instability among frames of the time-series OCT images and to reduce motion artifacts caused by external vibrations. Then, Welch’s method^26^^,^^37^ was applied to the time series of OCT intensity images to obtain the PSD of each pixel, as follows:

P=PWELCH(I), (1)

where P is the PSD sequence, and I is the pixel intensity in image sequences. The mean frequency is obtained from the following equation:

$$f_{\text{mean}}=\overset{M-1}{\underset{i=0}{\sum }}(P_{i}/\overset{M-1}{\underset{j=0}{\sum }}P_{j})\cdot f_{i},$$ (2)

where M denotes the length of the PSD sequence in the result of Eq. (1), and $P_{i}$ and $f_{i}$ are the $i_{\mathrm{th}}$ element and its frequency in the PSD sequence, respectively. Following this, we apply Gaussian filtering with a standard deviation of 3 and a kernel size of 3×3 to the average frequency image to remove aberrant pixel values caused by noise. Directly calculating the $f_{\text{mean}}$ for each pixel in the 512 frames of 660 (x)×660 (z) sized cross-sectional images is a time-consuming process. As shown in Fig. 2(a), the processing time could be reduced from ∼10 to ∼3  s by dividing the 660 (x)×660 (z) cross-section into four sub-datasets and parallel computing the $f_{\text{mean}}$ of the HCS cross-section, which effectively saved time when processing the 3D dynamic images of C3A-HCS. All image processing and analysis were performed using MATLAB (MathWorks Inc., Natick, Massachusetts, United States).

To improve the visualization of site-dependent cell viability in the HCS [Figs. 2(b) and 2(c)], different parameter values were calculated in the hue saturation value (HSV) channels [Fig. 2(d)] and mapped to the red-green-blue (RGB) space for pseudo-color display.^25^ The $f_{\text{mean}}$ ranges from 8 to 13 Hz, which we linearly scale to between 0 and 0.66 as the value for the hue channel, transitioning from red (low frequency) to blue (high frequency). The reciprocal of the frequency bandwidth serves as the saturation channel value, and the standard deviation of signal intensity serves as the intensity channel value. The resulting high-contrast, more intuitive pseudo-color images are shown in Fig. 2(e), where red-yellow corresponds to highly viable (proliferative) cell layers with lower motion frequency and higher magnitude of motion, green corresponds to low-viable cell layers with medium motion frequency, and blue corresponds to necrotic regions with higher motion frequency and lower magnitude of motion. The $f_{\text{mean}}$ can be calculated using Eq. (2), with the same results as in Fig. 2(d3). The standard deviation of the frequency was calculated by the power spectrum ($P_{i}$) and frequency ($f_{i}$), which is denoted as

$$f_{\mathrm{std}}=\overset{M-1}{\underset{i=0}{\sum }}(P_{i}/\overset{M-1}{\underset{j=0}{\sum }}P_{j})\cdot f_{i}^{2}-\overset{M-1}{\underset{k=0}{\sum }}[(P_{k}/\overset{M-1}{\underset{l=0}{\sum }}P_{l})f_{k}{]}^{2}.$$ (3)

**Fig. 3:**
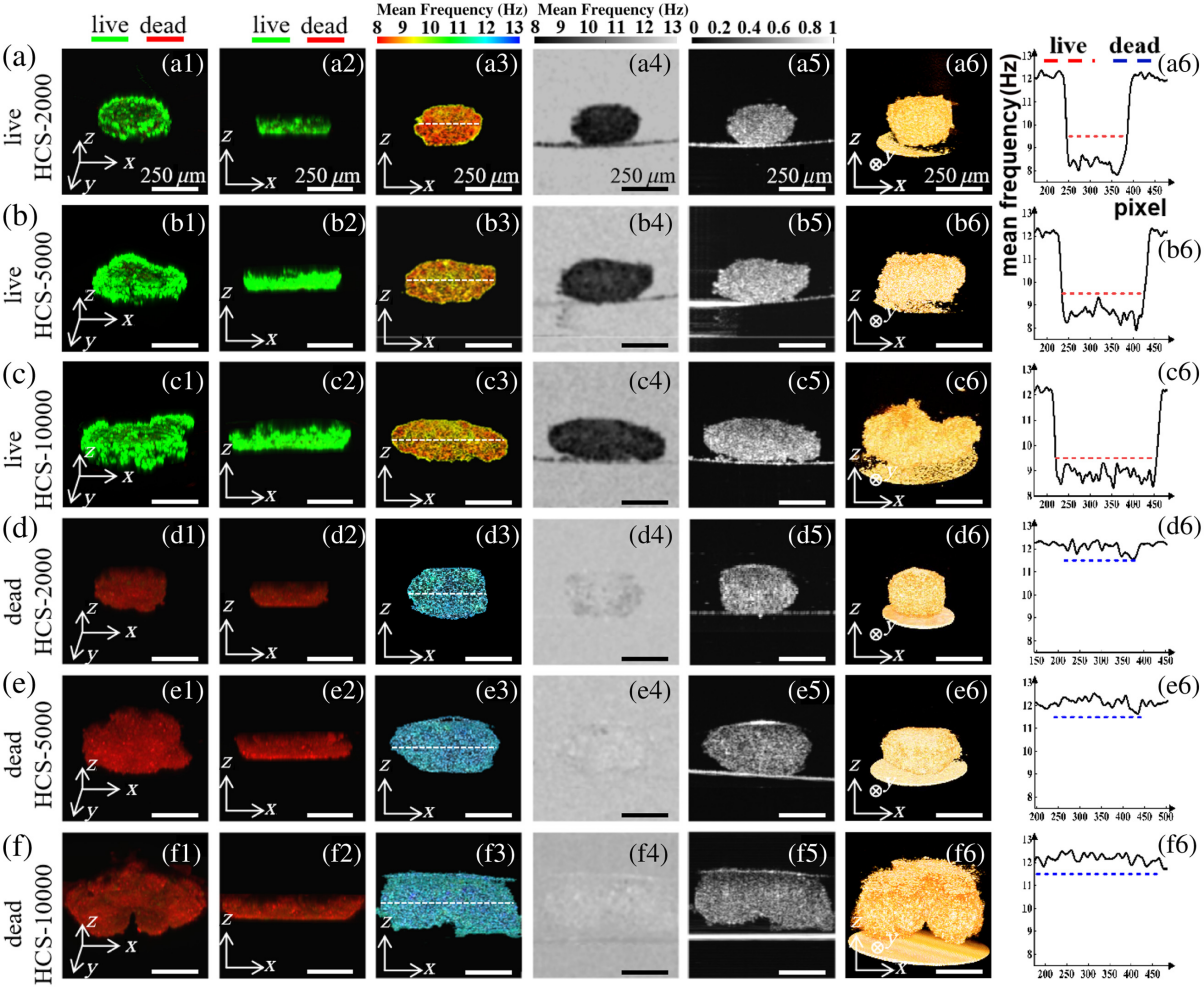
Comparison of D-OCT and CLSM images of fully viable C3A-HCS and fully necrotic C3A-HCS with different seeding numbers (HCS-2000, HCS-5000, and HCS-10000). (a)–(c) Results of fully viable C3A-HCS. (d)–(f) Results of fully necrotic C3A-HCS. The first and second columns show CLSM images in different directions: (a3), (b3), and (c3) show the high-viable regions in the D-OCT pseudo-color images obtained using mask segmentation, and (d3), (e3), and (f3) show the necrotic regions in the D-OCT pseudo-color images obtained using mask segmentation, where the white dashed lines indicate the penetration depth in CLSM. The fourth column shows the mean frequency image, the fifth column shows the static OCT intensity image, and the sixth column shows the 3D structure diagram of the 3D reconstructed HCS. The seventh column shows the $f_{\text{mean}}$ curve at the position of the white dashed line in the third column, where the red and blue dashed lines indicate high and low activities, respectively. Scale bar: 250  μm.

The speckle or background noise in the OCT intensity image showed a wide frequency band, which appeared black in the gray-scale saturation image [Fig. 2(d2)] after taking the reciprocal. The standard deviation, STD, of the time series OCT intensity image is calculated by

$$\mathrm{STD}=\sqrt{\frac{1}{N}\overset{N-1}{\underset{i=0}{\sum }}(I(t_{i})-\overset{¯}{I}{)}^{2}},$$ (4)

where N is the length of the image series, $I(t_{i})$ is the $i_{\mathrm{th}}$ image in the series, and $\overset{¯}{I}$ is the averaged image of the series. The resultant intensity image is shown in Fig. 2(d1), which is used to image the gray level of each pixel, with greater motion magnitude areas exhibiting a greater gray level. Figure 2(f) shows the mask for segmenting C3A-HCS to remove noise signals caused by the strong specular reflections from the concave-bottomed wells. It is reached using an empirical intensity threshold in Fig. 2(c). The two masks for segmenting the high-viable region [Fig. 2(g1)] and for low-viable region in the cross-section [Fig. 2(g3)] were created using the $f_{\text{mean}}$ threshold determined in Fig. 3 and intersecting with the mask of Fig. 2(f). It should be noted that a blue-green–colored circle existed on the outermost side of the pseudo-color D-OCT image [Fig. 2(e)] due to the interface reflection caused by the refractive index difference between the C3A-HCS edge and the culture medium during OCT mapping. This reflection led to pseudo-high-frequency signals at the C3A-HCS edge, which was mapped to the RGB space and displayed in blue-green. Therefore, the C3A-HCS edge was not a low-viable area. When using mask plate segmentation, the outermost region with fast backscattered light fluctuation can be removed, and true high activity region [Fig. 2(g2)] and low activity region [Fig. 2(g4)] can be obtained.

**Fig. 4:**
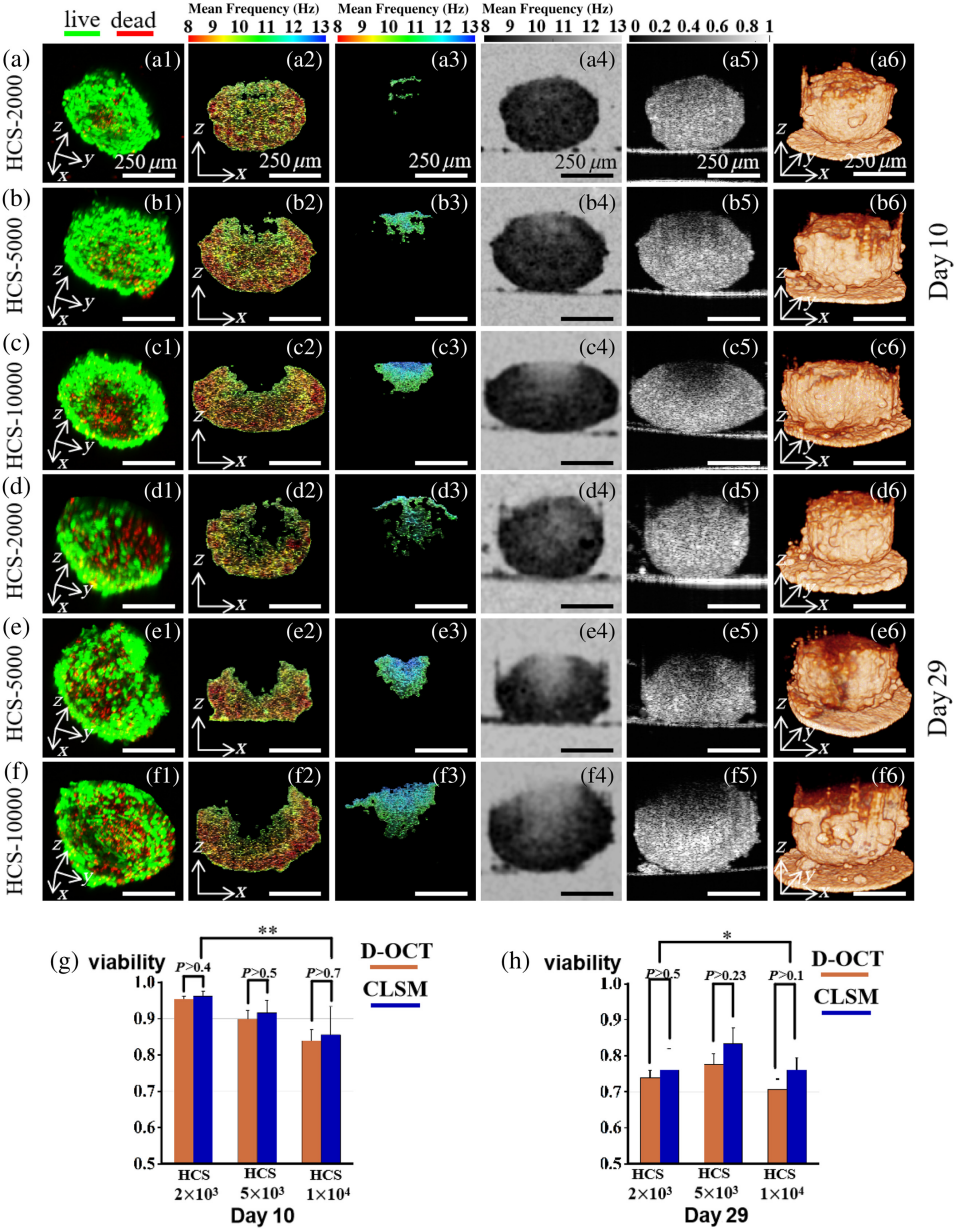
Comparison of D-OCT and CLSM images of C3A-HCS with different seeding numbers (HCS-2000, HCS-5000, and HCS-10000) and different culture days (days 8 and 17). (a)–c) Results of 10 days of culture. (d)–(f) Results of 29 days of culture. The first column shows the CLSM images, the second column shows the high-viable regions in the D-OCT pseudo-color images obtained using mask segmentation, the third column shows the necrotic regions in the D-OCT pseudo-color images obtained using mask segmentation, the fourth column shows the mean frequency image, the fifth column shows the static OCT intensity image, and the sixth column shows the 3D structure diagram of the 3D reconstructed HCS. (g) and (h) Bar charts of viability-D-OCT and viability-CLSM for C3A-HCS on days 10 and 29, respectively. Experimental values are expressed in mean ± standard error, n=3. Two-way ANOVA: *P<0.05, **P<0.01, and ***P<0.001. Scale bar: 250  μm.

### 2.4 Confocal Laser Scanning Microscopy and Fluorescence Microscopy Imaging

To verify the accuracy of the detection of C3A-HCS viability using the mean frequency-based D-OCT method, CLSM was used to detect the viability of stained C3A-HCS. Calcein acetoxymethyl was used for staining the viable cells,^7^ which is a hydrophobic compound that easily penetrates intact viable cells and appears green under an excitation wavelength of 525 nm. Propidium iodide was used for staining the necrotic cells, which cannot penetrate the cell membrane and can only bind to the deoxyribonucleic acid (DNA) of dead cells and appears red under an excitation wavelength of 593 nm.

CLSM imaging is usually applied in the optical sectioning fluorescence imaging of biological samples and can achieve multicolor colocalization imaging and reconstruct 3D scanning. The imaging system used here was a single-photon confocal microscope (A1 HD25, Nikon, Tokyo, Japan) with an imaging depth of ∼150  μm.^8^

### 2.5 Statistical Analysis

Three independent experiments were conducted with the experimental data presented as mean ± standard error. Imaging method (D-OCT and CLSM) and C3A-HCS samples (HCS-10000, HCS-5000, and HCS-2000) were used as the two factors for the two-way analysis of variance (ANOVA) test, with a significance level set at P<0.05 to indicate statistical significance.

## 3 Result

### 3.1 Multimethod Validation of C3A-HCS Viability Detection

Figure 3 illustrates the comparison of D-OCT and CLSM images of fully viable C3A-HCS and fully necrotic C3A-HCS with different seeding numbers. We regarded cell spheres on the first day of spheroid formation as fully viable, whereas those fixed with 4% PFA for 10 min were considered fully necrotic. The dynamic processing results of the central cross-section of the C3A-HCS were compared with the CLSM images. Notably, owing to the differences in imaging principles of the detection tools, CLSM images captured sections in the x−y horizontal direction, whereas SD-OCT images captured sections in the x−z vertical direction. However, the impact of such differences can be minimized by capturing C3A-HCS central sections.

As shown in Figs. 3(a1)–3f(1) and 3(a2)–3f(2), fully viable C3A-HCS appear completely green in CLSM fluorescence images, indicating a survival rate of 100%, whereas fully necrotic C3A-HCS show entirely red in CLSM fluorescence images, indicating a survival rate of 0%. As illustrated in Fig. 3(a3)–3f(3), the corresponding D-OCT results for fully viable C3A-HCS exhibit red-yellow, indicating high activity, whereas fully necrotic C3A-HCS show all blue, indicating low activity. The CLSM and D-OCT results serve as mutual references to each other.

Noticeable differences occur in the frequency of motion between viable and necrotic cells as demonstrated by Park et al.,^19^ through the observation of the intracellular dynamic activities of HeLa cells in different survival states. As shown in Figs. 3(a4)–3f(4) and 3(a6)–3f(6), the $f_{\text{mean}}$ across the entire x−z cross-section of fully viable C3A-HCS was evenly distributed at 8∼9.5  Hz, whereas the $f_{\text{mean}}$ across the entire x−z cross-section of fully necrotic C3A-HCS was evenly distributed at 11.5∼13  Hz. The $f_{\text{mean}}$ in the fully viable C3A-HCS and fully necrotic C3A-HCS showed an apparent difference. Based on the lower limit of $f_{\text{mean}}$ of necrotic cells and the upper limit of $f_{\text{mean}}$ of highly viable cells, the mean value $f_{\text{mean}}=10.5\;\mathrm{Hz}$ could be used as a threshold for the segmentation of the region with different cell viability states. We also calculated the average value of $f_{\text{mean}}$ over the entire x−z cross-section for fully viable C3A-HCS and fully necrotic C3A-HCS in Fig. 3 (see Table S2 in the Supplementary Material). The average value of $f_{\text{mean}}$ for fully viable C3A-HCS is 8.8±0.2  Hz, and for fully necrotic C3A-HCS, it is 12.2±0.4  Hz. The average value of $f_{\text{mean}}$ of cross-section was obtained by summing the $f_{\text{mean}}$ values of all pixels and dividing by the number of pixels. We also analyzed the power spectral density curves of the fully viable C3A-HCS and fully necrotic C3A-HCS (see Fig. S2 in the Supplementary Material). We also presented the power spectral density curves of fully viable C3A-HCS and fully necrotic C3A-HCS at different sampling frequencies (see Fig. S3 in the Supplementary Material).

Figure 4 shows the mutual validation results of the C3A-HCS cell viability pseudo-color images based on power spectrum analysis of the time series of OCT intensity images and CLSM images. As the central region of the spheroids was not in direct contact with the nutrients, so they show low activity and even necrosis due to the lack of nutrients during the culture progression.^38^ The phenomenon can be reflected by the D-OCT pseudo-color image near the C3A-HCS center in the x−z cross-section. If there is no necrosis in the entire C3A-HCS, then it would not be detected in cross-sections [Fig. 4(a)]. In case of low activity or necrotic cells in the center of the C3A-HCS, it can be detected in both cross-sections close to the center of the spheroids [Fig. 4(f)].

Significant differences in C3A-HCS necrosis were observed among the C3A-HCSs with different initial cell seeding numbers cultured until day 10 (group B) in three batches. The CLSM image of HCS-2000 was overall green, indicating highly viable cells within the spheroid [Fig. 4(a1)]. The D-OCT image was overall red-yellow, indicating a low motion frequency, hence suggesting highly viable cells within the spheroid. Both CLSM and D-OCT images indicated that the HCS-2000 were highly viable on day 10 of the culture. Meanwhile, the CLSM and D-OCT images of the HCS-5000 showed very few low-viable cells on day 10, with red fluorescence representing dead cells in the CLSM image [Fig. 4(b1)] and blue-green color representing low-viable cells in the D-OCT image [Fig. 4(b3)]. A comparison of CLSM and D-OCT results showed that D-OCT could identify necrotic cell regions, and the regions detected by D-OCT and CLSM were consistent in terms of depth. The HCS-10000 was larger than the HCS-2000 and HCS-5000, making it more prone to nutrient deficiency in the central region by day 10. In the HCS-10000 [Fig. 4(c)], the number of necrotic cells inside increased significantly, with necrotic cells concentrated at the center and the upper surface of the spheroid [Fig. 4(c3)]. Compared with day 10, the number of necrotic cells in C3A-HCSs with different cell seeding numbers continued to increase when cultured until day 29 (group C). Among them, the reduction in cell viability was most apparent in the HCS-2000. As seen from the static OCT images of Figs. 4(a4)–4(f4), the cell sphere thickness was already more than 250  μm after C3A-HCS culture up to day 10. The CLSM imaging captured the 3D live/dead cell distribution of C3A-HCS within a depth of 0 to 150  μm, but it was unable to obtain the entire 3D image of the spheroids due to the limited penetration depth.

To further verify the accuracy of D-OCT viability detection, the cell viability in D-OCT and CLSM images were quantitatively analyzed. Cell spheroid viability was defined as the pixel ratio of live cells (green fluorescence) to total cells (sum of green and red fluorescence) in CLSM (viability-CLSM) images; the ratio of the live cell area segmented by D-OCT to the total cell area in the cross-section was termed as the spheroid viability detected by D-OCT (viability-D-OCT). Bar charts of spheroid viability values for different detection methods were plotted to quantify the relationship of viability detection between D-OCT and CLSM. Figures 4(g) and 4(h) represent bar charts for viability-D-OCT and viability-CLSM, respectively, obtained from three different types of HCS in a three-batch experiment. The results showed that there was no significant difference between the two detection methods (P>0.05). However, significant differences were observed among different samples (P<0.01), with decreased viability values using both methods as HCS volume increased. Further explanations for these findings are provided in Sec. 4. Through qualitative and quantitative comparisons with CLSM images, it can be concluded that our proposed D-OCT method accurately performs HCS viability detection.

### 3.2 3D Site-Dependent Cell Viability of C3A-HCS

**Fig. 5:**
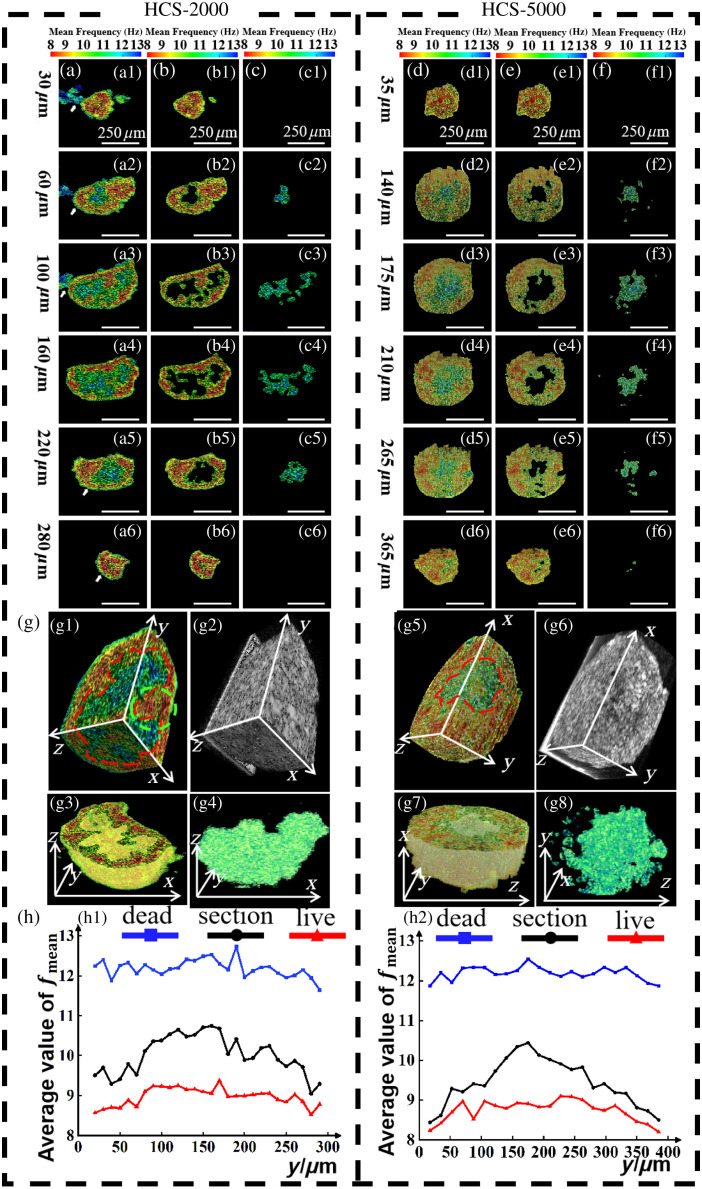
2D and 3D structure of the HCS with different seeding numbers (HCS-2000 and HCS-5000) obtained by D-OCT on day 29. (a) and (d) Dynamic pseudo-color image. (b) and (e) Highly viable regions segmented using a mask. (c) and (f) Necrotic regions segmented using a mask. (g) Dynamic/static OCT 3D reconstruction of HCS, with (g1) and (g5) site-dependent cell viability of the three different cross-sections within HCS, with the red dashed lines delineating the core necrotic and the outer highly viable region and green dashed circle delineating the highly active regions within the spheroids; (g2) and (g6) static OCT 3D structure of HCS; (g3) and (g7) 3D structure of the highly viable cell layers; and (g4) and (g8) 3D structure of the necrotic cell layers. (h) Average value of $f_{\text{mean}}$ curve of the cross-sections at different depths from the HCS surface. The white arrows point to the misclassified areas removed using the masking method, which we discuss in Sec. 4. Scale bar: 250  μm.

Figure 5 shows different seeding numbers (HCS-2000 and HCS-5000) of C3A-HCS. The HCS-2000 is ellipsoidal with maximum and minimum diameters of 450 and 300  μm, respectively, whereas HCS-5000 is spherical with a diameter of 450  μm. At the beginning of the culture, C3A-HCS exhibited a smaller volume, and its internal and surface cells could obtain a sufficient nutrient supply. With increasing culture time, the spheroids gradually grew in size, leading to the gradual emergence of distinct low-viable or even necrotic areas inside them. Figures 5(a) and 5(d) shows the dynamic pseudo-color image of C3A-HCS, with (a1)–(a6) and (d1)–(d6) representing cross-sections at different distances from the C3A-HCS surface (30, 60, 100, 160, 220, and 280 and 35, 140, 175, 210, 265, and 365  μm). The dynamic pseudo-color image was obtained by dynamically processing the different cross-sections. Cross-sections close to the spheroid surface [Figs. 5(a1), 5(a6), 5(d1), and 5(d6)] were almost all high-viable cell regions, with a calculated $f_{\text{mean}}$ of ∼9.5  Hz, showing yellow-red color. Cross-sections near the spheroid center [Figs. 5(a3), 5(a4), 5(d3), and 5(d4)] had larger necrotic areas, with a calculated $f_{\text{mean}}$ of ∼10.5  Hz, showing blue-green color. Using a mask to separate high-viable [Figs. 5(b) and 5(e)] and necrotic regions [Figs. 5(c) and 5(f)] in different cross-sections, the outer high-viable cell layers [Figs. 5(g3) and 5(g7)] and the core necrotic cell layers [Figs. 5(g7) and 5(g8)] were reconstructed. The necrotic cell layers had an irregular 3D structure, and its boundary could be separated [red dashed line in Fig. 5(f1)]. Compared with the static OCT 3D structure image [Figs. 5(g2) and 5(g6)], the D-OCT 3D pseudo-color image of C3A-HCS [Figs. 5(g1) and 5(g5)] displayed the distribution of live and dead cells inside C3A-HCS with high contrast (see Video 2 and Video 3 for the 3D D-OCT images of C3A-HCS; see Video 4 and Video 5 for the 3D structure of the highly viable cell layers; see Video 6 and Video 7 for the 3D structure of the necrotic cell layers). Comparison of the x−z, x−y, and y−z cross-sections showed that the boundary between the highly viable and necrotic regions of C3A-HCS became more intuitive, enabling more accurate identification of highly active regions within the spheroids [green dashed circle in Fig. 5(g1)]. Such detail is difficult to detect using the fluorescence assay method.

The average value of $f_{\text{mean}}$ of the cross-section was obtained by summing the $f_{\text{mean}}$ values of all pixels and dividing by the number of pixels, for the quantitative analysis of the overall distribution of cellular viability in the C3A-HCS. Figure 5(h) is the quantification result of the average value of $f_{\text{mean}}$ of each cross-section in C3A-HCS, and we obtained a narrow 95% confidence interval band (see Fig. S1 and Table S1 in the Supplementary Material). The blue curve represents the segmented necrotic region, with the average values of $f_{\text{mean}}$ of the cross-section maintained at 8 to 9.5 Hz, whereas the red curve represents the segmented high-viable region, with cross-sectional average values of $f_{\text{mean}}$ maintained at 11.5 to 13 Hz. The black curve represents the average values of the $f_{\text{mean}}$ of the entire cross-section. As the cross-section approaches the two outer surfaces of the C3A-HCS (0 to 50  μm and 250 to 300  μm), the average values of the $f_{\text{mean}}$ became lower, whereas the proportion of low-viable cells and the average values of the $f_{\text{mean}}$ would increase as it approaches the spheroid center. This quantitatively demonstrated that necrosis was most severe at the center of the spheroid, which is consistent with the biological characteristics of C3A-HCS.^4^^,^^38^

### 3.3 Analysis of the Longitudinal Necrotic Process of the C3A-HCS During the Growth Cycle

**Fig. 6:**
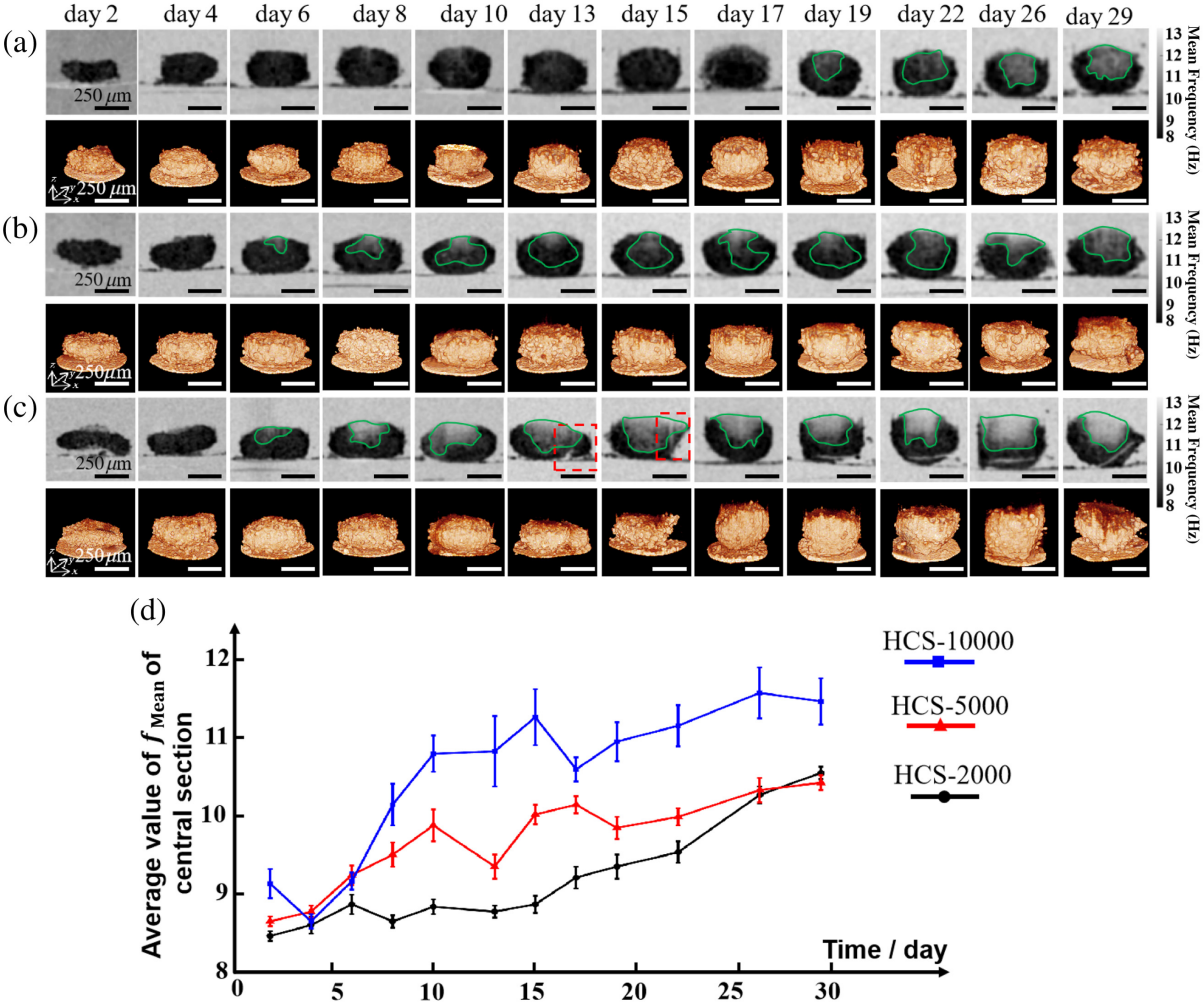
Continuous monitoring of tissue activity and 3D structural changes in the C3A-HCS. (a)–(c) $f_{\text{mean}}$ of the central cross-sections and 3D structural images of the HCS-2000, HCS-5000, and HCS-10000, respectively. (d) Variation curves of the average value of $f_{\text{mean}}$ of the central cross-sections of the three C3A-HCSs. The green solid line box represents the necrotic part of the cell sphere, and the red dashed line box represents a part of the cell sphere that detached from the original spheroid. Scale bar: 250  μm.

Figure 6 shows the longitudinal observation and quantitative analysis of the necrotic process in C3A-HCS with three different cell seeding numbers over 30 days using OCT, including 3D structures and changes in the $f_{\text{mean}}$ of HCS central cross-sections. In the early stages of the culture, the HCS-2000, HCS-5000, and HCS-10000 exhibited irregular flat disc-shaped or ellipsoidal 3D structures. As the culture days increased, the diameters of the three C3A-HCS types slightly decreased to a stable value and the height increased, making the HCSs become spherical.

The HCS-2000 cell spheroids were smaller in volume, and the internal cells in the HCS-2000 could obtain sufficient nutrients and oxygen supply [Fig. 6(a)]. The cell viability changes were relatively small during the first 19 days of cultivation, until day 26 when a noticeable necrotic region appeared in the cell spheroids, with a low-activity gray-white area appearing at the center. The $f_{\text{mean}}$ for the central cross-section of HCS-5000 cell spheroids remained in a growth phase for the first 10 days, and decreased on days 13 and 19 [Fig. 6(b)]. This may be due to the position change of the microspheres by replacing the culture medium every 48 h, resulting in a shift in the collected central cross-section and capturing more sections with a higher proportion of live cells in the ellipsoidal HCS. However, the overall changes were consistent with the expected growth trend. The $f_{\text{mean}}$ image of the central cross-section of HCS-10000 cell spheroids exhibited significant changes on day 8 with the appearance of a noticeable necrotic gray-white region (green solid line box on day 8), which continued to grow until day 15 and decreased on day 17 [Fig. 6(c)]. This is because a part of the HCS-10000 cell spheroids has detached from the original spheroid [red dashed line boxes on days 13 and 15 in Fig. 6(c)]. The left major part continued to increase $f_{\text{mean}}$ in the central cross-section during the subsequent cultivation process, with the necrotic portion inside gradually becoming larger.

By plotting the $f_{\text{mean}}$ of the C3A-HCS central cross-section as a function of cultivation days, the dynamic changes in tissue can be observed more quantitatively. Different color curves were used to show the tissue activity changes of the three types of C3A-HCS in the three-batch experiment [Fig. 6(d)]. In the early stage of cultivation (first 6 days), the cell spheroids of HCS-2000, HCS-5000, and HCS-10000 exhibited similar tissue activity (the average values of $f_{\text{mean}}$ of the central cross-sections are between 8 and 9.5 Hz); as the cultivation days increased, the average values of $f_{\text{mean}}$ of the central cross-sections of the three types of HCS cell spheroids gradually increased and exhibited different trends of slope changes, indicating an increase in the necrotic areas in the three types of HCS cell spheroids, but the rate of increase differed.

## 4 Discussion

A multilayered structure of proliferating and necrotic cells in the 3D cell spheroids appears when incubation time is increased, or the drug is applied. Effective detection of this necrotic region in the core is crucial for obtaining accurate drug toxicity and efficacy data.^4^ In this study, a D-OCT imaging method was employed based on SD-OCT devices and power spectral analysis for the quantitative assessment and visualization of site-dependent cell viability within large-volume 3D biological samples. The activity frequency of viable C3A cells within HCS stayed below 20 Hz, and the 48 kHz A-scan and 56 fps B-scan acquisition speeds were sufficient to distinguish the cells with different activities. To improve the 2D/3D imaging speed of the D-OCT, the acquired B-scan was divided into four or nine equal parts for parallel calculation of the $f_{\text{mean}}$, which greatly decreased the data processing time at a single cross-section from 10 to 3 s. In contrast to the ATP activity detection method,^10^^,^^11^ which requires disruption of spheroid structure to determine overall tissue activity, the D-OCT method can accurately detect the activity status of cells in different layers within C3A-HCS samples without disrupting the spheroid structure. To quantitatively analyze the frequency of cellular activity within biological samples, the imaging contrast was enhanced by the mean frequency image of cellular activity, achieving high-precision detection, segmentation, and quantification of different cell viability regions based on cellular activity frequencies. Using a masking method to segment and display cells with different activities,^19^ the proportion of live cells was quantitatively analyzed to assess tissue activity. The “viability” is defined as the ratio of live cell number to total cell number. Currently, due to the limitations of D-OCT volume imaging speed, only the viability of the cross-section in C3A-HCS was calculated. In the dynamic pseudo-color display, both frequency and amplitude of the OCT time series intensity signal were used for the visualization of site-dependent cell viability. Compared with the RGB mapping method based on directly segmenting the mean frequency, the color transition was more natural, and the representation of cellular dynamics information was richer.

The dynamic OCT imaging method proposed in this paper can detect necrotic regions within 3D tissue samples, as shown in the red dashed box in Fig. 5(f1); necrosis within such samples is difficult to detect by CLSM detection methods because of the limited imaging depth.^7^^,^^8^ In this paper, we found that the viability-DOCT of the same type of C3A-HCS would be slightly lower than the viability-CLSM [Figs. 4(g) and 4(h)], and with the increase of incubation time, the difference between viability-DOCT and viability-CLSM of the same type of C3A-HCS increases. This is probably due to the fact that the cell viability on the surface of the cell spheres is higher than that inside, whereas CLSM mainly detects cell viability in the superficial layer of the sample. As the culture time increases, the volume of C3A-HCS increases, the detectable fraction of CLSM decreases as a proportion of the whole sphere, and the quantified tissue activity stays above the actual value. For the proposed 3D D-OCT detection method, viability-D-OCT of the entire HCS spheroid could be calculated if a higher OCT detection speed can be achieved. Thus, it enables higher accuracy than that of viability-CLSM.

As Fig. 5(f1) precisely describes the boundaries of the necrotic region within the sphere, the accurate display of the cellular viability of each layer within the cell sphere, can provide new research ideas for drug testing based on 3D *in vitro* models. For example, drug diffusion is closely related to the treatment of solid tumors, and the size and diffusion rate of drug molecules will directly affect the drug effect on tumor cells, which in turn affects the efficacy of tumor drugs.^4^^,^^39^^–^^41^ Figure 6 shows the results of the long-term longitudinal observation of C3A-HCS, indicating that the dynamic method can be used to study the impact of the initial seeding number on the growth state of cell spheroids. This helps determine the optimal seeding number of cell spheroids for drug screening. The D-OCT method proposed in this study can accurately describe the cellular viability state of each layer after drug application, which can provide a new tool for studying the spatial pattern of drug diffusion and screening for more effective anticancer drugs with better diffusion properties.^13^ The longitudinal monitoring results of HCS necrosis (Fig. 6) show that D-OCT without affecting the long-term continuous culture of samples accurately detects cellular activity within the samples and quantitatively analyzes the frequency of cellular activity in different regions, demonstrating its value in probing 3D tissue growth and development patterns, with promising future applications in more complex engineered tissues such as organoids.

It is important to note that the low-viable cell areas located at the junction of the culture medium were removed using the masking method as indicated by the white arrows in Figs. 2 and 5. This low-viability cell area was caused by the change in the refractive index at the contact part between the cell mass and the culture medium. The mask used was further processed from Fig. 2(g3). Specifically, we evaluated the connected regions on the HCS boundary in Fig. 2(g3) and removed any pixels that were more than five pixels away from the boundary of the necrotic areas. Compared with Fig. 5(a), the removal of the outermost blue region in Fig. 5(b) shows higher cell viability in the outermost blue region than in the core region, yielding a more biologically consistent cell activity distribution image in the pseudo-color display, but this mask removal of the outermost misclassification reduces the quantitative value of tissue viability. To verify the accuracy of D-OCT activity detection, we compared the x−z cross-section of D-OCT with the x−y horizontal plane of CLSM, which was an alternative method adopted due to the slower 3D activity detection speed of D-OCT. In future studies, the speed of 3D D-OCT imaging would be increased in terms of algorithm implementation, hardware acceleration, and data acquisition protocols,^24^ making real-time 3D D-OCT imaging possible, which is of great significance for the promotion and application of this tool.

## 5 Conclusion

In this study, a D-OCT imaging method based on SD-OCT and power spectrum analysis was employed to study the dynamics of cellular spheres. The cells in C3A-HCS were found to be active mainly in the range of 8 to 13 Hz by D-OCT detection, and then, the live and dead cells were distinguished by the mean frequency of cellular activity, and the high-viable and necrotic regions were segmented based on the mask and the tissue activity of the cell spheres was quantified. The HSV color mapping method enhanced the visualization effect and segmentation accuracy of viability differences and exhibited a more readable viability transition display effect than the direct frequency segmentation method for RGB color mapping. The accuracy of D-OCT in detecting cell viability was validated by conducting comparative imaging analysis using D-OCT and CLSM on fully viable C3A-HCS and fully necrotic C3A-HCS at different cell inoculation amounts of HCS-2000, HCS-5000, and HCS-10000. The comparative imaging analysis of D-OCT and CLSM for C3A-HCS with different cell inoculation amounts at various time points also supported this finding. It also shows the advantage of the D-OCT method to detect site-dependent cell viability in 3D samples. The analysis of cell activities in the C3A-HCS in 3D shows the potential of the D-OCT imaging method to study the state of cellular viability across layers within the three-dimensional model; the results of longitudinal observations of C3A-HCS over long periods of time validate the value of D-OCT for non-destructive observation of changes in tissue activity. Overall, the D-OCT method proposed in this paper can provide a new non-destructive quantitative detection tool for the fields of tissue engineering, drug screening, and organoids.

## 6 Appendix: Video Captions

The following videos are mentioned in the text:

**Video 1** Real-time video of the OCT intensity map of C3A-HCS (MP4, 3.69 MB [URL: https://doi.org/10.1117/1.JBO.30.3.035003.s1]).

**Video 2** 3D D-OCT maps of C3A-HCS (MPG, 6.13 MB [URL: https://doi.org/10.1117/1.JBO.30.3.035003.s2]).

**Video 3** 3D D-OCT maps of C3A-HCS (MPG, 5.3 MB [URL: https://doi.org/10.1117/1.JBO.30.3.035003.s3]).

**Video 4** 3D structure of the highly viable cell layers (MPG, 4.2 MB [URL: https://doi.org/10.1117/1.JBO.30.3.035003.s4]).

**Video 5** 3D structure of the highly viable cell layers (MPG, 3.3 MB [URL: https://doi.org/10.1117/1.JBO.30.3.035003.s5]).

**Video 6** 3D structure of the necrotic cell layers (MPG, 2.53 MB [URL: https://doi.org/10.1117/1.JBO.30.3.035003.s6]).

**Video 7** 3D structure of the necrotic cell layers (MPG, 2.33 MB [URL: https://doi.org/10.1117/1.JBO.30.3.035003.s7]).

## Supplementary Material

10.1117/1.JBO.30.3.035003.s01

10.1117/1.JBO.30.3.035003.s1

10.1117/1.JBO.30.3.035003.s2

10.1117/1.JBO.30.3.035003.s3

10.1117/1.JBO.30.3.035003.s4

10.1117/1.JBO.30.3.035003.s5

10.1117/1.JBO.30.3.035003.s6

10.1117/1.JBO.30.3.035003.s7

## Data Availability

Code and data underlying the results presented in this paper are not publicly available at this time but may be obtained from the authors upon reasonable request.
