# Supplementary material for: Quantitative evaluation of the site-dependent cell viability in three-dimensional hepatocyte spheroids based on dynamic optical coherence tomography
Source: J Biomed Opt. 2025 Mar 14;30(3):035003. doi: 10.1117/1.JBO.30.3.035003 (PMC11934154; doi:10.1117/1.JBO.30.3.035003)
Supplement: Supplementary file 1 [file JBO_030_035003_SD001.pdf]

Quantitative evaluation of the site-dependent cell viability in 3D hepatocyte spheroids based on dynamic optical coherence tomography: supplemental document

1 Average value of f_{mean} curve with 95% confidence interval bands of the cross-sections at different depths from the HCS surface.

The average value of f_{mean} of cross-section was obtained by summing the f_{mean} values of all pixels and dividing by the number of pixels, for the quantitative analysis of the overall distribution of cellular viability in the C3A-HCS. The average value of f_{mean} curve with 95% confidence interval bands of the cross-sections at different depths from the HCS surface are shown in Figure S1.The blue curve represents the segmented necrotic region, with the average values of f_{mean} of the cross-section maintained at 8-9.5 Hz, whereas the red curve represents the segmented high-viable region, with cross-sectional average values of f_{mean} maintained at 11.5-13 Hz. The black curve represents the average values of the f_{mean} of the entire cross-section.

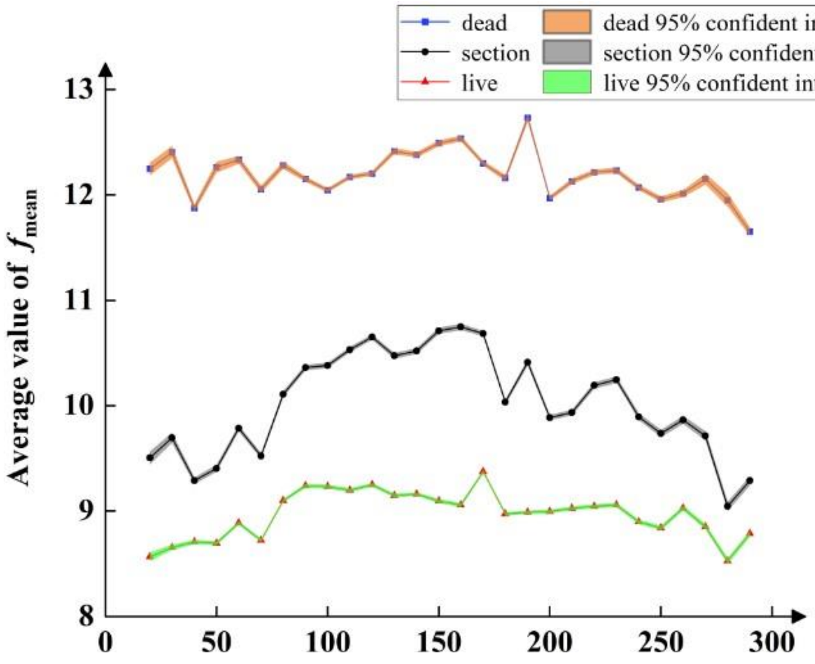

Figure S1. The average value of f_{mean} curve with 95% confidence interval bands of the cross-sections at different depths from the HCS surface

2 Specific values of the mean and 95% confidence interval upper and lower limits

The specific values of the mean and 95% confidence interval upper and lower limits are shown in Table S1

Table S1. The specific values of the mean and 95% confidence interval upper and lower limits

Depth (μm)	Dead mean	Dead upper limits	Dead lower limits	Section mean	Section upper limits	Section lower limits	Live mean	Live upper limits	Live lower limits
20	12.2467	12.3087	12.1861	9.5055	9.5674	9.4436	8.5664	8.6089	8.5238
30	12.4054	12.4648	12.3474	9.6964	9.7467	9.6474	8.6554	8.6825	8.6283
40	11.8765	11.9139	11.8378	9.2888	9.3210	9.2565	8.7070	8.7289	8.6851
50	12.2635	12.3164	12.2106	9.4062	9.4397	9.3726	8.6954	8.7160	8.6760
60	12.3332	12.3719	12.2945	9.7867	9.8177	9.7545	8.8863	8.9044	8.8682
70	12.0545	12.0907	12.0171	9.5236	9.5519	9.4952	8.7225	8.7405	8.7044
80	12.2816	12.3190	12.2455	10.1092	10.1376	10.0795	9.0991	9.1159	9.0824
90	12.1500	12.1771	12.1229	10.3621	10.3904	10.3350	9.2398	9.2591	9.2204
100	12.0442	12.0661	12.0223	10.3827	10.4085	10.3569	9.2320	9.2514	9.2127
110	12.1706	12.1926	12.1487	10.5323	10.5594	10.5052	9.1998	9.2191	9.1791
120	12.2029	12.2274	12.1784	10.6510	10.6768	10.6239	9.2501	9.2681	9.2307
130	12.4144	12.4454	12.3848	10.4743	10.5027	10.4446	9.1482	9.1675	9.1301
140	12.3809	12.4093	12.3525	10.5194	10.5478	10.4911	9.1611	9.1791	9.1430
150	12.4906	12.5176	12.4622	10.7104	10.7413	10.6807	9.0966	9.1172	9.0759
160	12.5344	12.5641	12.5060	10.7478	10.7787	10.7168	9.0579	9.0798	9.0372
170	12.3009	12.3241	12.2777	10.6846	10.7104	10.6588	9.3739	9.3907	9.3571
180	12.1603	12.1926	12.1294	10.0344	10.0615	10.0060	8.9740	8.9934	8.9560
190	12.7305	12.7705	12.6918	10.4124	10.4459	10.3801	8.9882	9.0063	8.9714
200	11.9694	11.9939	11.9462	9.8873	9.9144	9.8615	8.9972	9.0153	8.9792
210	12.1294	12.1590	12.1010	9.9364	9.9634	9.9080	9.0269	9.0450	9.0088
220	12.2158	12.2429	12.1874	10.1931	10.2240	10.1621	9.0463	9.0669	9.0256
230	12.2313	12.2584	12.2055	10.2460	10.2782	10.2124	9.0566	9.0772	9.0346
240	12.0700	12.0971	12.0442	9.8951	9.9286	9.8615	8.8979	8.9198	8.8747
250	11.9591	11.9887	11.9294	9.7377	9.7725	9.7029	8.8399	8.8631	8.8166
260	12.0133	12.0494	11.9771	9.8654	9.9041	9.8280	9.0282	9.0527	9.0050
270	12.1500	12.2016	12.0984	9.7132	9.7583	9.6693	8.8502	8.8786	8.8231
280	11.9513	12.0171	11.8868	9.0450	9.0901	9.0011	8.5264	8.5561	8.4967
290	11.6508	11.6946	11.6069	9.2888	9.3339	9.2449	8.7870	8.8179	8.7560

3 The average value of f_{mean} of cross-section for fully viable C3A-HCS and fully necrotic C3A-HCS with different seeding numbers (HCS-2000, HCS-5000, and HCS-10000).

The average value of f_{mean} over the entire x-z cross-section for fully viable C3A-HCS and fully necrotic C3A-HCS with different seeding numbers (HCS-2000, HCS-5000, and HCS-10000) are shown in Table.S2. Three independent experiments were conducted for each seeding number. The average value of f_{mean} for fully viable C3A-HCS is 8.8 ± 0.2 Hz, and for fully necrotic C3A-HCS is 12.2 ± 0.4 Hz. The average value of f_{mean} of cross-section was obtained by summing the f_{mean} values of all pixels and dividing by the number of pixels.

Table S2. The average value of f_{mean} over the entire x-z cross-section for fully viable C3A-HCS and fully necrotic C3A-HCS

fully viable C3A-HCS-2000	fully viable C3A-HCS-5000	fully viable C3A-HCS-10000	fully viable C3A-HCS mean	fully viable C3A-HCS STD	fully necrotic C3A-HCS-2000	fully necrotic C3A-HCS-5000	fully necrotic C3A-HCS-10000	fully necrotic C3A-HCS mean	fully necrotic C3A-HCS STD
8.6632	8.7724	9.0804			11.7404	12.3200	12.6616		
8.3888	8.8648	8.9152	8.7774	0.1997	11.5472	12.6308	12.3984	12.1915	0.4265
8.6996	8.6856	8.9264			11.7600	12.5944	12.0708		

4 Power spectral density curve and time-varying spectral signal intensity curve of C3A-HCS.

Figure S2 shows the power spectral density curves and the time-varying spectral signal intensity curves for viable, necrotic, and low activity regions. From the power spectral density curve, it can be observed that the peak frequency of the viable C3A-HCS is around 0.1 Hz, suggesting it has a relatively low movement frequency. In contrast, the peak of the power spectral density curve for necrotic C3A-HCS is in the range of 19-22 Hz, indicating a higher movement frequency. Furthermore, we analyzed the low activity regions within the C3A-HCS, and its power spectral density curve displays two peaks, one around 0.1 Hz and another within the 19-22 Hz range.

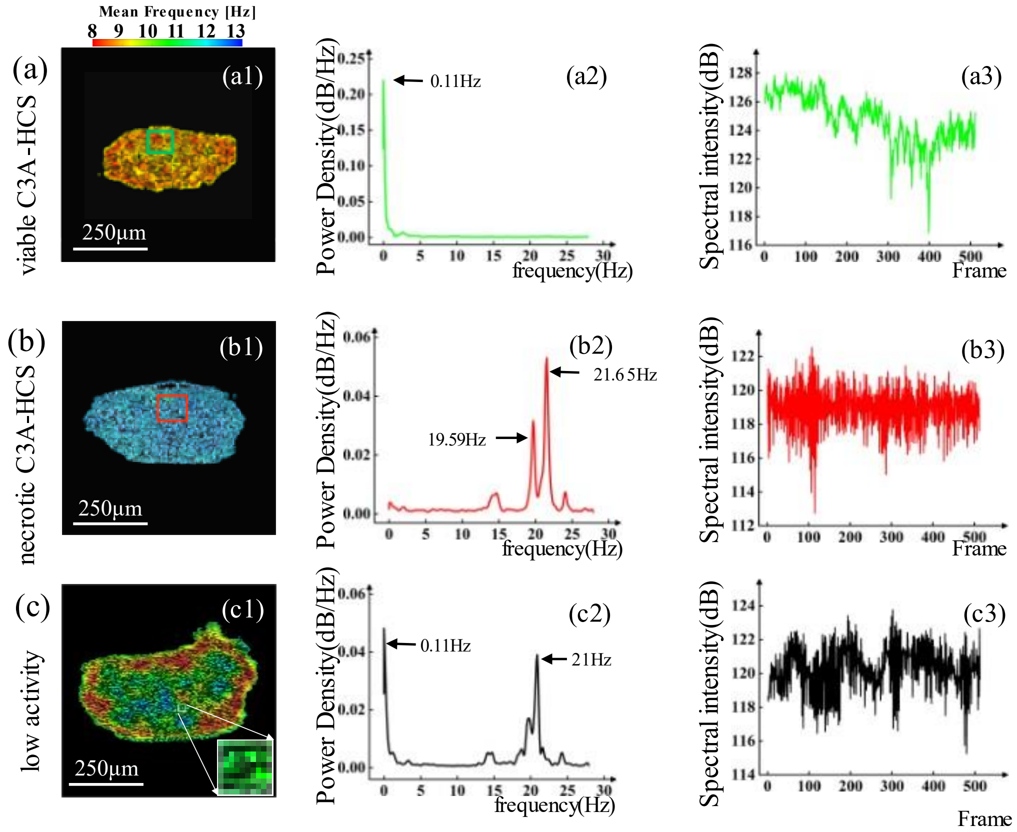

Figure S2. Power spectral density curve and time-varying spectral signal intensity curve of C3A-HCS.(a) the results of fully viable C3A-HCS. (b) the results of fully necrotic C3A-HCS. (c) the results of low activity C3A-HCS. The first columns shows dynamic pseudo-color image. The second columns shows the power spectral density curve. The third columns shows the time-varying spectral signal intensity curve. The green, red, and white implementation boxes represent the selected pixel range. Each pixel within the box has a power spectral density curve and a time-varying spectral signal intensity curve. We averaged the curves of all the pixels to obtain fig(a2)-(c2) and fig(a3)-(c3). Scale bar: 250μm.

5 The power spectral density curves for fully viable C3A-HCS and fully necrotic C3A-HCS at B-scan acquisition speeds of 56 frames/s and 110 frames/s, respectively.

Figure S3 shows the power spectral density curves for fully viable C3A-HCS and fully necrotic C3A-HCS at B-scan acquisition speeds of 56 frames/s and 110 frames/s, respectively. We collected data at an A-scan rate of 76 kHz, with each B-scan image containing 343 A-scans, resulting in dynamic data with a B-scan rate of 110 frames/s. It can be observed that the power spectral density peak for the fully viable C3A-HCS is around 0.1 Hz in both cases. At 110 Hz, the power spectral density peak appears at 0.21 Hz due to the minimum frequency resolution being 0.21 Hz at this sampling rate. Simultaneously, the power spectral density peaks for the fully necrotic C3A-HC are consistently within the 19-22 Hz range. This indicates that the sampling frequency does

not affect the value of f_{mean}.

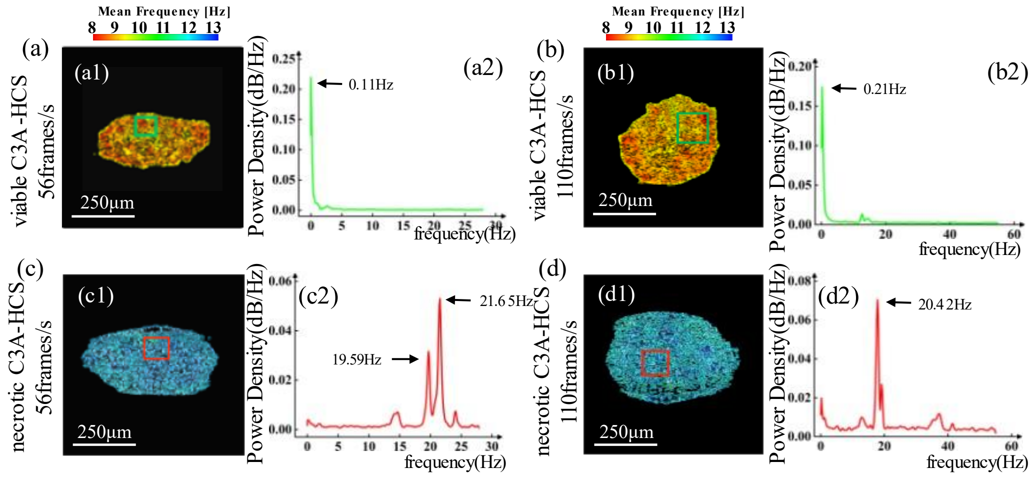

Figure S3. Power spectral density curves for fully viable C3A-HCS and fully necrotic C3A-HCS at B-scan acquisition speeds of 56 frames/s and 110 frames/s, respectively. (a) (a1) Dynamic pseudo-color image of fully viable C3A-HCS at a B-scan acquisition rate of 56 frames/s, and (a2) the corresponding power spectral density curve. (b) (b1) Dynamic pseudo-color image of fully viable C3A-HCS at a B-scan acquisition rate of 110 frames/s, and (b2) the corresponding power spectral density curve. (c) (c1) Dynamic pseudo-color image of fully necrotic C3A-HCS at a B-scan acquisition rate of 56 frames/s, and (c2) the corresponding power spectral density curve. (d) (d1) Dynamic pseudo-color image of fully necrotic C3A-HCS at a B-scan acquisition rate of 110 frames/s, and (d2) the corresponding power spectral density curve. The green and red implementation boxes represent the selected pixel range. Each pixel within the box has a power spectral density curve. We averaged the curves of all the pixels to obtain fig(a2)-(d2). Scale bar: 250μm.
